# Purtscher-Like Retinopathy Secondary to an Appendiceal Neuroendocrine Neoplasm Complicated by a Periappendiceal Abscess

**DOI:** 10.7759/cureus.85752

**Published:** 2025-06-11

**Authors:** Bartosz Skulimowski, Slawomir Liberski, Danuta Nikratowicz, Anna Gotz-Wieckowska

**Affiliations:** 1 Ophthalmology, Poznan University of Medical Sciences, Poznań, POL

**Keywords:** appendiceal neuroendocrine neoplasm, cotton wool spots, purtscher flecken, purtscher-like retinopathy, retinal microvasculopathy

## Abstract

Purtscher-like retinopathy (PLR) is a rare retinal microvasculopathy that is induced by non-traumatic systemic illnesses. We describe the case of a woman in her 70s who was diagnosed with PLR, ultimately linked to an appendiceal neuroendocrine neoplasm (ANEN) complicated by a periappendiceal abscess. The patient presented with sudden, unilateral, painless visual impairment. Initial examination revealed Purtscher flecken (PF) and cotton-wool spots (CWS) in the right eye, with corresponding hyperreflectivity and swelling of the inner retinal layers on optical coherence tomography (OCT). Systemic evaluation revealed elevated D-dimer levels: 547 ng/mL fibrinogen equivalent units (FEU) (normal <500 ng/mL FEU), and subsequent imaging identified a periappendiceal infiltrate. Laparoscopic appendectomy confirmed the presence of a well-differentiated grade 3 ANEN. Postoperative follow-up demonstrated regression of retinal findings and partial visual improvement (of BCVA from 1.4 logMAR to 0.5 logMAR in the affected eye). However, she presented a scotoma in the visual field of the right eye. The follow-up OCT showed localized inner retinal atrophy corresponding to prior ischemic changes. To our knowledge, this is the first reported case linking PLR to ANEN or appendicitis. This case highlights the importance of systemic evaluation in patients with PLR, particularly when typical aetiologies are excluded.

## Introduction

Purtscher-like retinopathy (PLR) is a rare retinal vascular condition, first described in the 1990s to delineate non-traumatic cases resembling Purtscher’s retinopathy (PR). Typical retinal findings include cotton-wool spots (CWS), Purtscher flecken (PF), and retinal haemorrhages [1]. In contrast to PR, which arises secondary to traumatic injury, PLR is of non-traumatic origin and can be caused by various systemic conditions, the most common being acute pancreatitis and systemic lupus erythematosus (SLE). Other, less prevalent PLR causes include systemic vasculitis, pre-eclampsia, and fat or amniotic fluid embolisms. PLR typically manifests as a sudden, painless decrease in visual acuity [2]. CWS are the most prevalent clinical sign and are present in 93% of PR and PLR cases. Intraretinal haemorrhages (IRH) and PF were found in approximately 65% of documented cases. PF is considered to be pathognomonic for both PR and PLR [3].

The condition typically arises in the context of systemic diseases promoting hypercoagulability. Available data suggest that the aetiology of PLR is based on the activation of both plasma and platelet prothrombotic factors but also other potential proembolic factors, such as aggregated leukocytes, fibrin, or fat droplets, occurring in the setting of systemic inflammation and leading to embolization in small vessels of the retina [2-4]. This likely causes an ischemic injury to the inner retina. Neoplasms are well known for their prothrombotic potential. Several cases of PLR have been reported in association with malignant neoplasms. Documented malignancy-related causes include pancreatic adenocarcinoma, gastric carcinoma, and hematologic malignancies such as multiple myeloma and leukemia [2,5]. To date, however, PLR has not been described in association with appendiceal neuroendocrine neoplasms (ANEN). While cases of PLR associated with systemic inflammatory abdominal conditions such as ischemic colitis have been described, the spectrum of gastrointestinal causes remains narrow and poorly documented [6].

## Case presentation

A Caucasian woman in her 70s presented to the Ophthalmology Outpatient Clinic with a three-day history of blurred vision in her right eye (OD). She described the visual disturbances as “dark-grey snowflakes on glass.” One month earlier, she had experienced a transient episode of “kaleidoscopic”-colored lights in the same eye.

The patient reported generalized weakness and loss of appetite over the past six months, which had led to noticeable weight loss. For the past two years, she had also experienced a pulling sensation in the right inguinal region. An abdominal ultrasound and laboratory tests, including erythrocyte sedimentation rate (ESR) and C-reactive protein (CRP), performed six months before presentation, showed no significant abnormalities. A chest X-ray performed three days before was unremarkable.

Her medical history included ischemic heart disease, for which she had undergone percutaneous coronary intervention (PCI) after ischemic changes were detected on an exercise ECG. As part of her treatment, she had been taking 75 mg of acetylsalicylic acid (ASA) daily. She also had hyperlipidaemia and a history of cholecystectomy due to gallbladder stones. The patient was also a chronic smoker.

**Figure 1:**
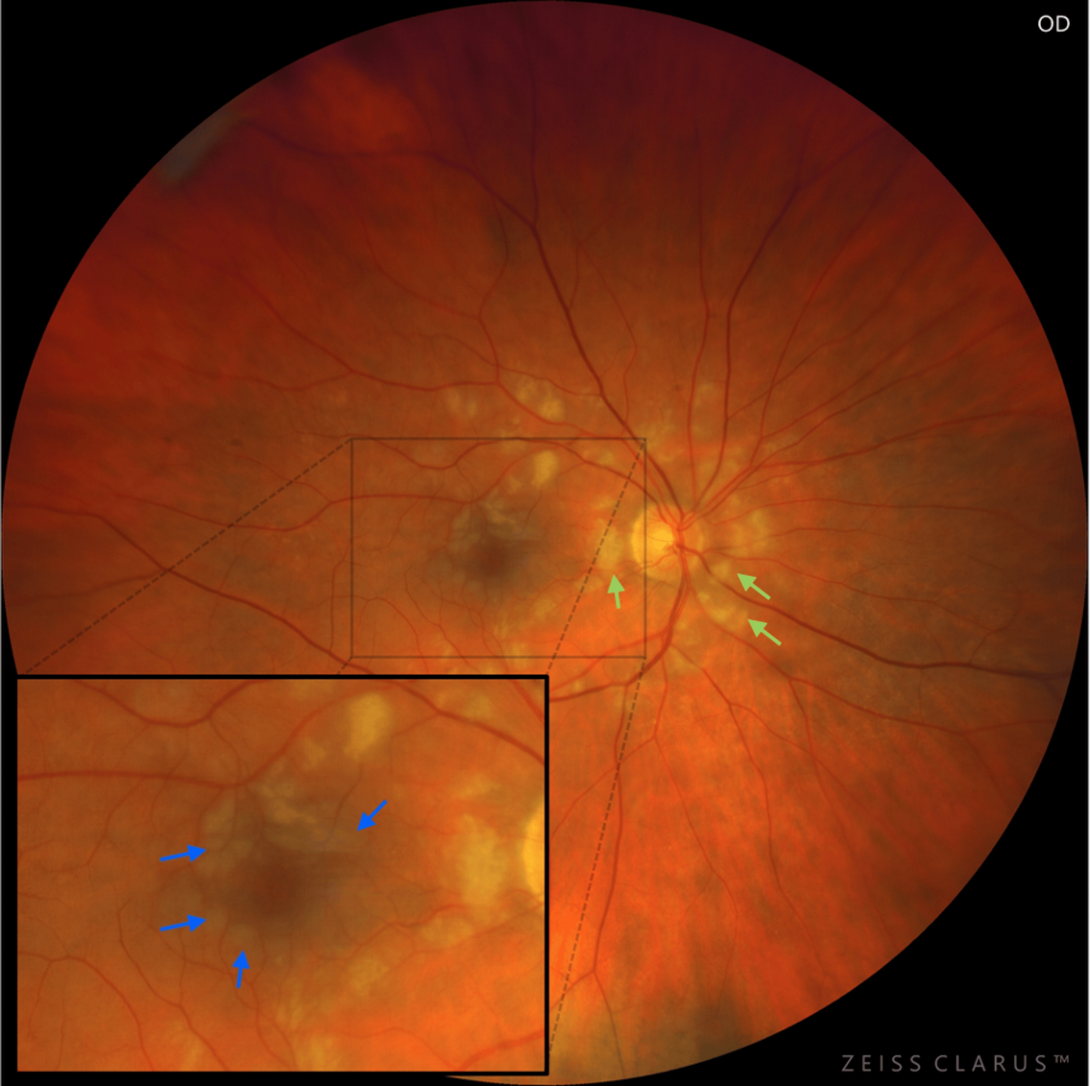
Fundus photograph of the right eye taken at initial presentation. The photograph shows PF with characteristic lesion-free areas in the immediate vicinity of the retinal vessels (indicated by blue arrows), particularly prominent within the macula. The optic disc and vascular arcades are surrounded by CWS (indicated by green arrows). Narrowing of the retinal vessels can also be observed.

**Figure 2:**
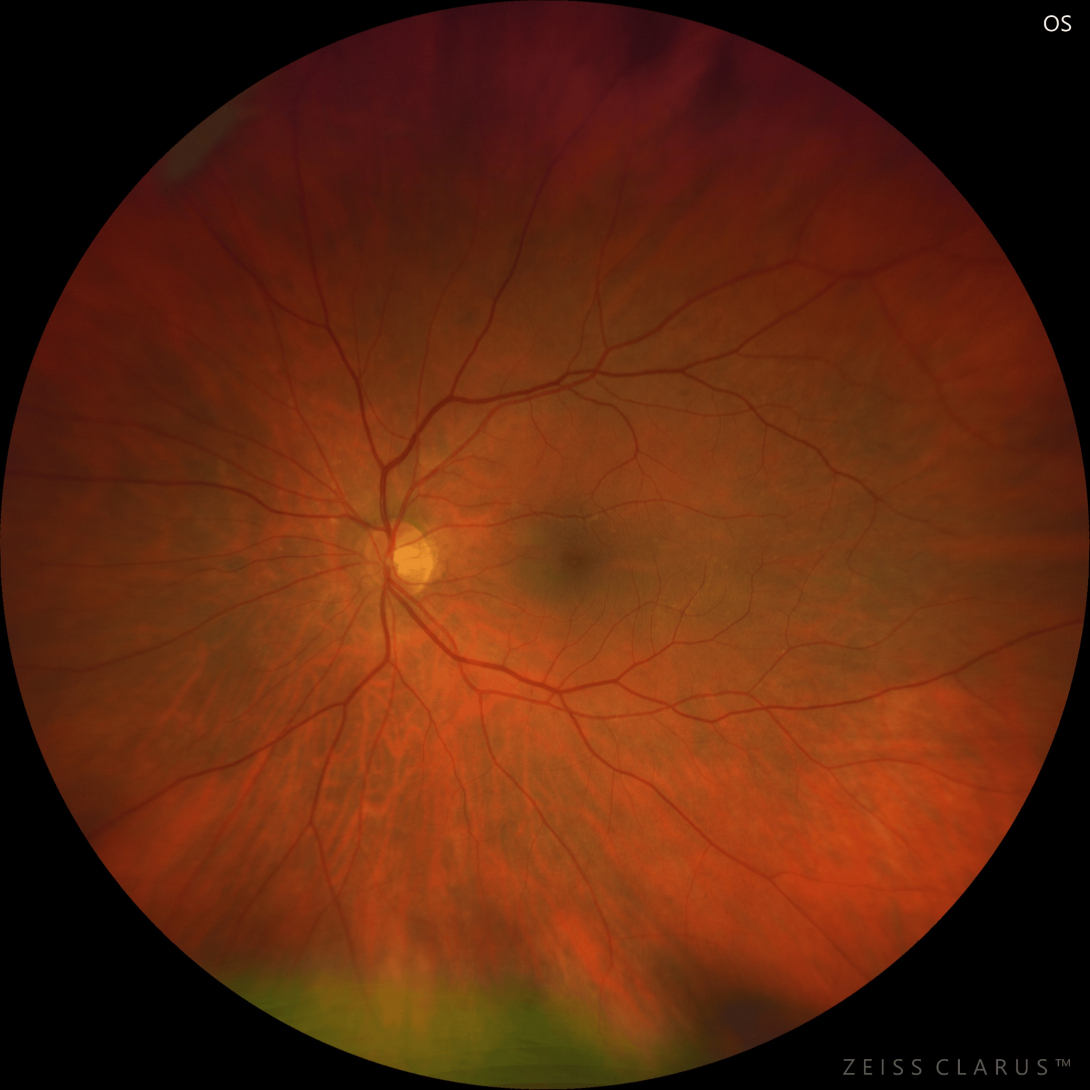
Fundus photograph of the left eye taken at initial presentation. The photograph shows the posterior pole of the left eye at the time of initial presentation. The narrowing of the retinal vessels can be observed, as it was in the right eye, but the fundus is otherwise normal.

**Figure 3:**
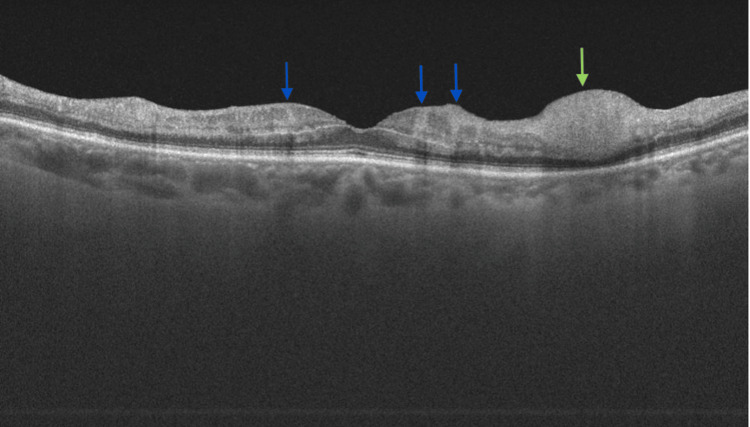
Optical coherence tomography scan of the macula of the right eye, obtained at the time of initial presentation. The scan shows localized hyperreflectivity of the inner retinal layers. Visible are the junctions inner nuclear layer/outer plexiform layer (INL/OPL) and OPL/outer nuclear layer (ONL) junctions, correlating with PF (indicated with blue arrows). Focal swelling of the retinal nerve fibre layer (RNFL) corresponds to cotton-wool spots (CWS) (indicated with green arrows).

**Figure 4:**
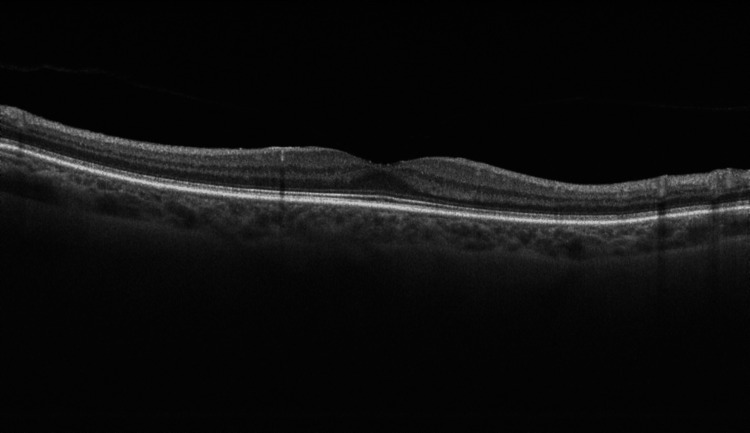
Optical coherence tomography scan of the macula of the left eye, obtained at the time of initial presentation. The OCT scan of the left eye shows normal macular architecture. The foveal contour is preserved, with normal layering of the retinal structures.

At presentation, her refractive error was: +2.00 DS / -0.50 DC × 3° in the OD and +1.75 DS in the OS. Her best corrected visual acuity (BCVA) was 1.4 logMAR in the OD, and 0.00 logMAR in the left eye (OS). In the Snellen's near vision test performed with a +3.00 DS near addition, her vision in the OD was very poor, corresponding to 2.4 logMAR. Near vision in the OS was normal, corresponding to 0.00 logMAR at 40 cm. Intraocular pressure (IOP) was 18 mmHg in both eyes. Anterior segment examination of both eyes showed no signs of inflammation or other abnormalities, except for lens opalescence in both eyes. Fundoscopic examination revealed multiple diffuse white fluffy lesions - cotton-wool spots (CWS) - in the posterior pole of the OD, accompanied by retinal vessel narrowing. Additionally, greyish spots confined by retinal vessels were noted in the macula-consistent with Purtscher flecken (PF) (Figure 1). The OS showed no abnormalities (Figure 2). Optical coherence tomography (OCT) of the OD macula demonstrated hyperreflectivity and blurring of the inner retinal layers. Junctions between the inner nuclear layer (INL) and the outer plexiform layer (OPL), as well as between the OPL and the outer nuclear layer (ONL), were visible and correlated with the presence of PF. Focal swelling of the retinal nerve fibre layer (RNFL) corresponded with CWS (Figure 3). The macular OCT of the OS was unremarkable (Figure 4).

**Figure 5:**
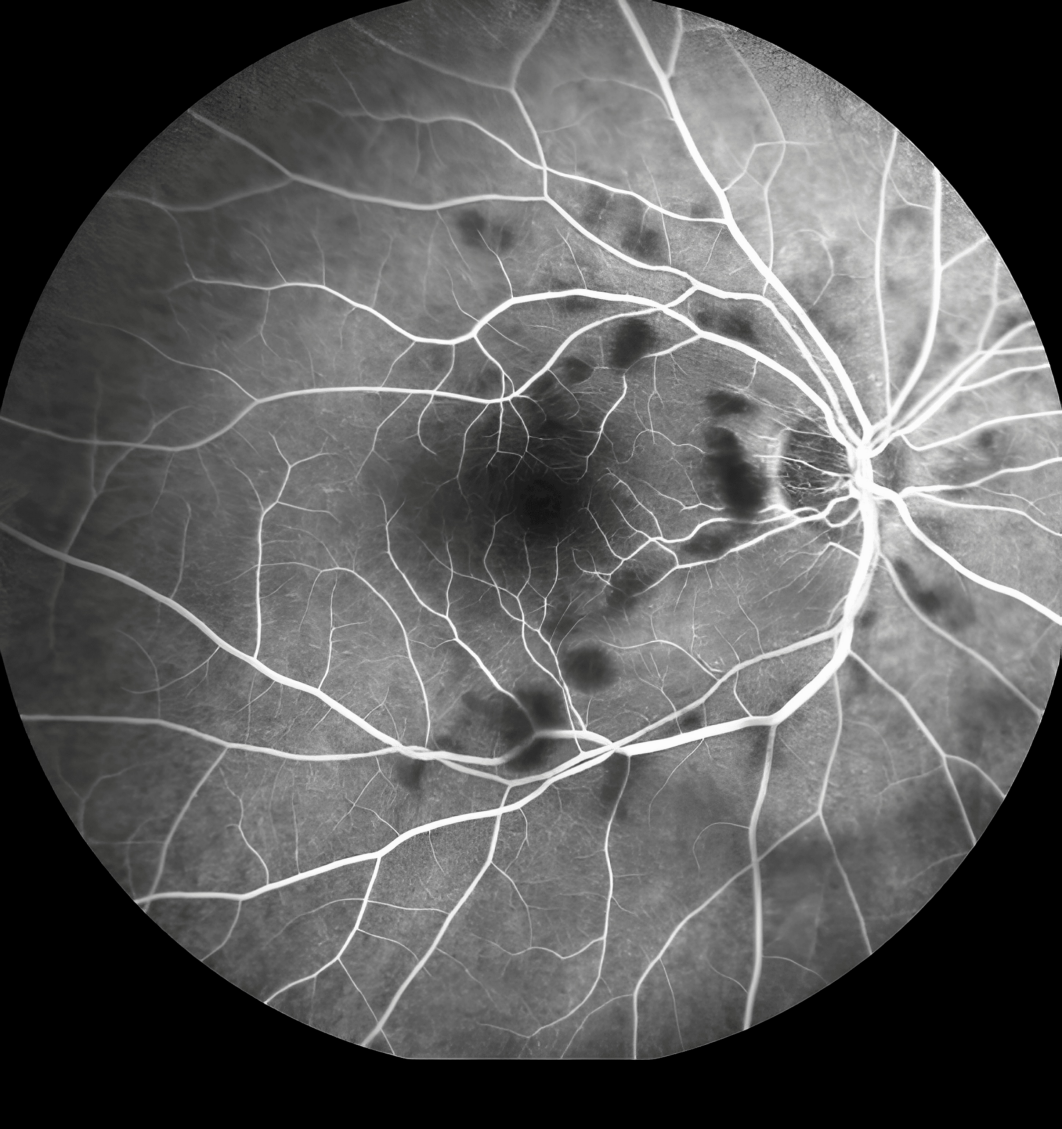
Late-phase fluorescein angiography of the right eye. The scan shows hypofluorescent areas corresponding to the greyish foci seen on the color fundus photograph. These findings are indicative of perfusion abnormalities.

Late-phase fluorescein angiography (FA) revealed hypofluorescent areas in the projection of the greyish foci visible on the color photograph that suggested perfusion abnormalities (Figure 5). A subtle leakage is visible in the nasal part of the macula, with no signs of macular neovascularization (MNV).

Given the suspected systemic cause of the retinal lesions, laboratory tests were ordered. Morphology, CRP, fasting plasma glucose, ESR, and rheumatoid factor (RF) were within normal range. The results showed a slightly elevated D-dimer level of 547 ng/mL fibrinogen equivalent units (FEU) (normal <500 ng/mL FEU). Serological tests for the following pathogens were performed: hepatitis B virus (HBV), human immunodeficiency virus (HIV), cytomegalovirus (CMV), Epstein-Barr virus (EBV), Treponema pallidum, and Toxoplasma gondii. They showed no evidence of an active infection. There were no abnormalities on abdominal ultrasound and chest X-ray.

These findings raised a strong suspicion that PLR was the most likely explanation for the patient’s clinical presentation. In the search for an underlying cause of PLR, she was referred for further outpatient evaluation at a surgical reference centre and scheduled for regular ophthalmology follow-up. The surgical referral was based on the presence of chronic right inguinal pain and weight loss, which raised the possibility of abdominal or pelvic pathologies. No pharmacological treatment was initiated at that time.

**Figure 6:**
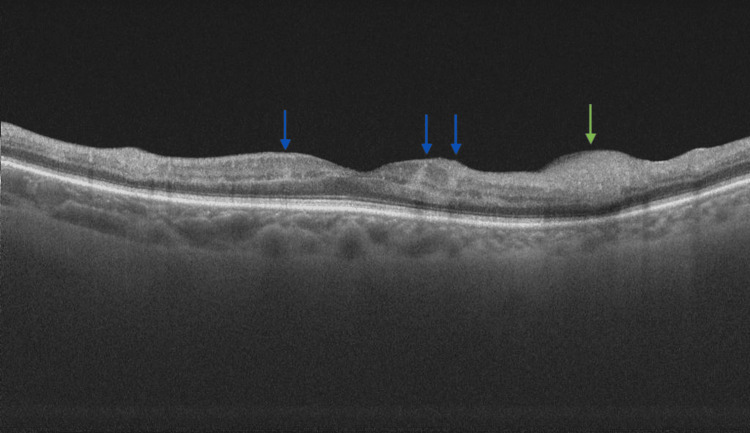
Optical coherence tomography scan of the macula of the right eye two weeks after the initial presentation. The scan shows alleviation of the focal swelling of the RNFL (indicated with green arrow) and a decrease in hyperreflectivity of the inner retinal layers (marked with blue arrows).

**Figure 7:**
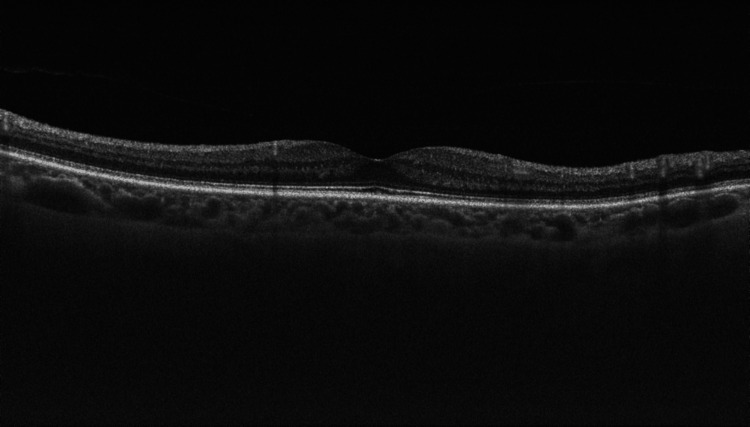
Optical coherence tomography (OCT) scan of the macula of the left eye two weeks after the initial presentation. The OCT scan of the left eye shows normal macular architecture. The foveal contour is preserved, with normal layering of the retinal structures.

At a planned follow-up examination seven days after the initial presentation, the BCVA of the OD improved to 1.0 logMAR, while the BCVA of the OS remained at 0.0 logMAR. IOP was within the normal range in both eyes. The retinal lesions in the OD persisted, with macular OCT findings remaining unchanged (Figure 6). The OS remained free of pathological changes (Figure 7). Two weeks after the initial presentation, the retinal changes in the OD were still present, but appeared more subtle, and the BCVA of the OD slightly improved to 0.7 logMAR.

**Figure 8:**
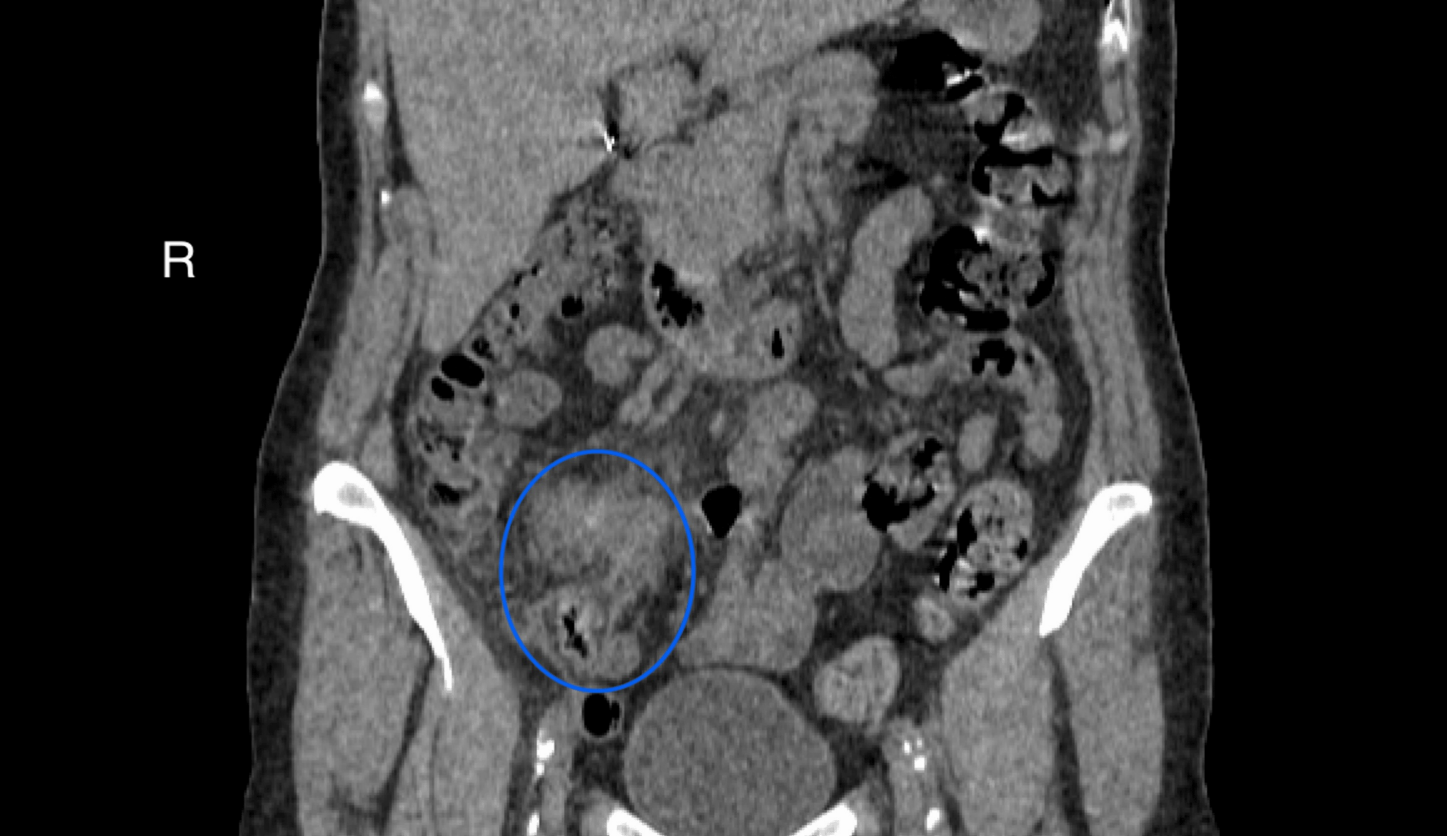
Non-contrast abdominal computed tomography (CT) scan in the coronal plane. The scan demonstrates a thickened appendix surrounded by an isodense region consistent with inflammatory infiltration. This complex is further encased by pericecal fat stranding, indicative of localized inflammatory changes.

**Figure 9:**
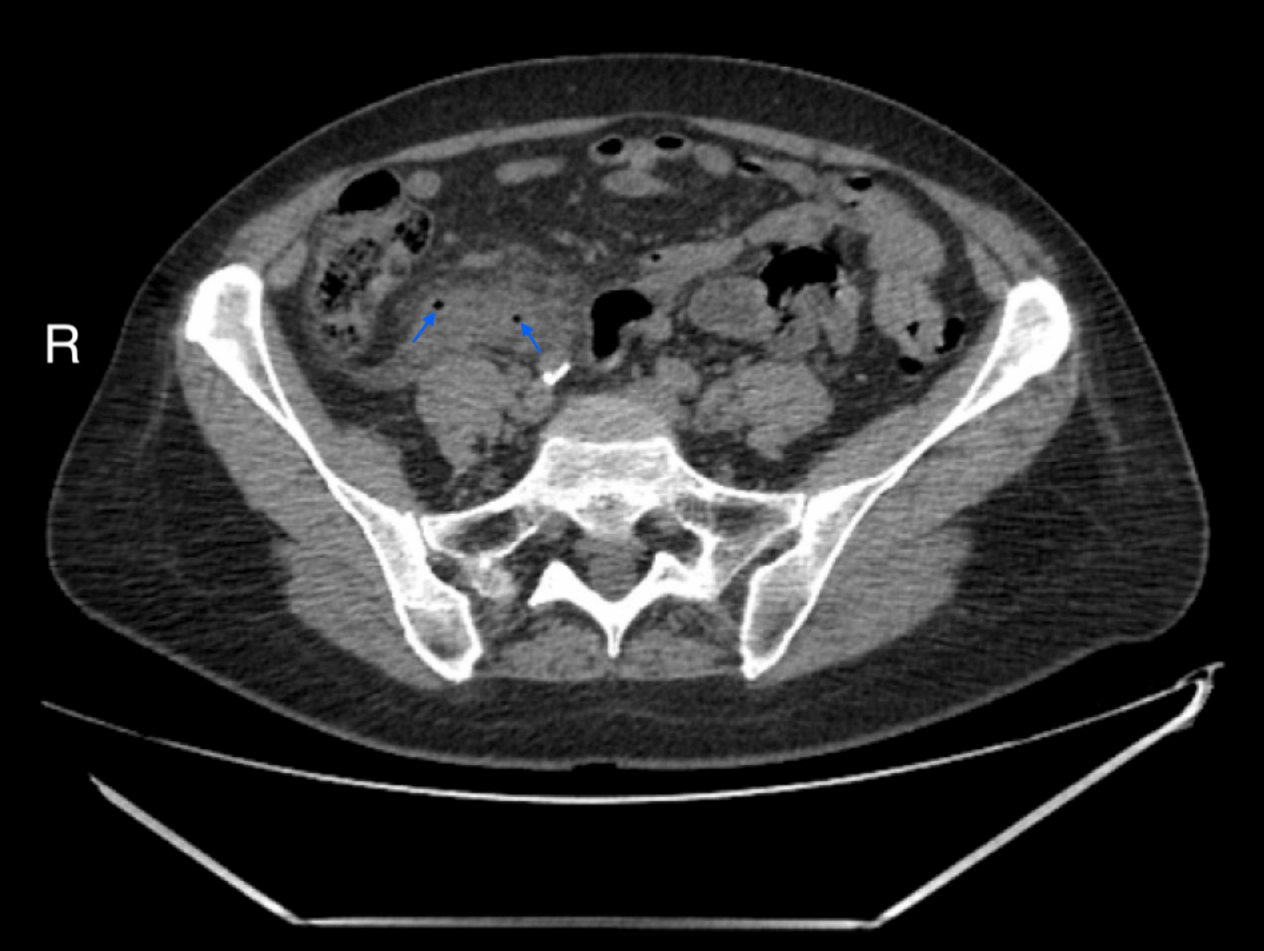
Non-contrast abdominal computed tomography (CT) in the axial plane. Axial CT sections reveal inflammatory infiltration in the right iliac fossa, accompanied by gas bubbles (indicated by arrows), suggestive of abscess formation.

About six weeks after the initial presentation, the patient experienced an episode of acute pain in the right groin. An abdominal ultrasound revealed a suspected inflammatory infiltrate in the right iliac fossa. Non-contrast abdominal computed tomography (CT) confirmed a suspected periappendiceal inflammatory infiltrate (Figures 8-9). The patient was admitted to the general surgery department. Intravenous antibiotic therapy, including metronidazole and ceftriaxone, was initiated, leading to clinical improvement. The patient was scheduled for elective surgery.

She underwent a laparoscopic residual appendectomy, with drainage of a periappendiceal abscess and lavage of the peritoneal cavity. Intraoperatively, plastron appendicitis was diagnosed. Histopathological examination of the resected appendix revealed a highly differentiated, grade 3 ANEN that infiltrated the entire thickness of the appendicular wall and periappendiceal fat tissue.

**Figure 10:**
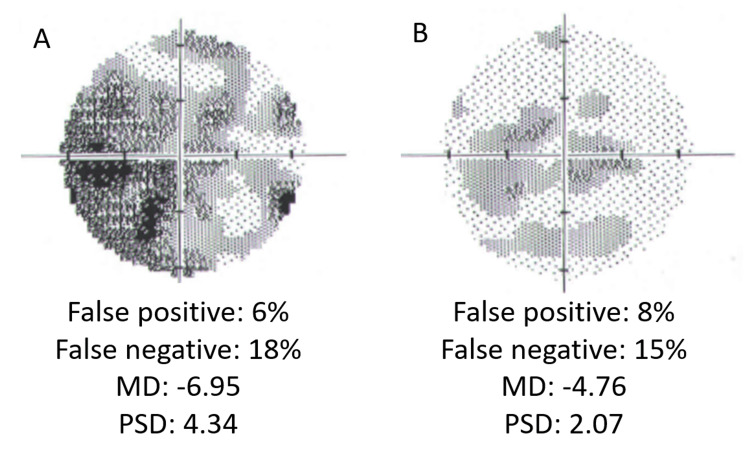
The results of a visual field examination using a Humphrey field analyzer. The Humphrey visual field (30-2) shows visual field defects in the right eye (A), the most pronounced in the inferior nasal quadrant, and mild sensitivity reduction in the visual field of the left eye (B). MD = mean deviation; PSD = pattern standard deviation

**Figure 11:**
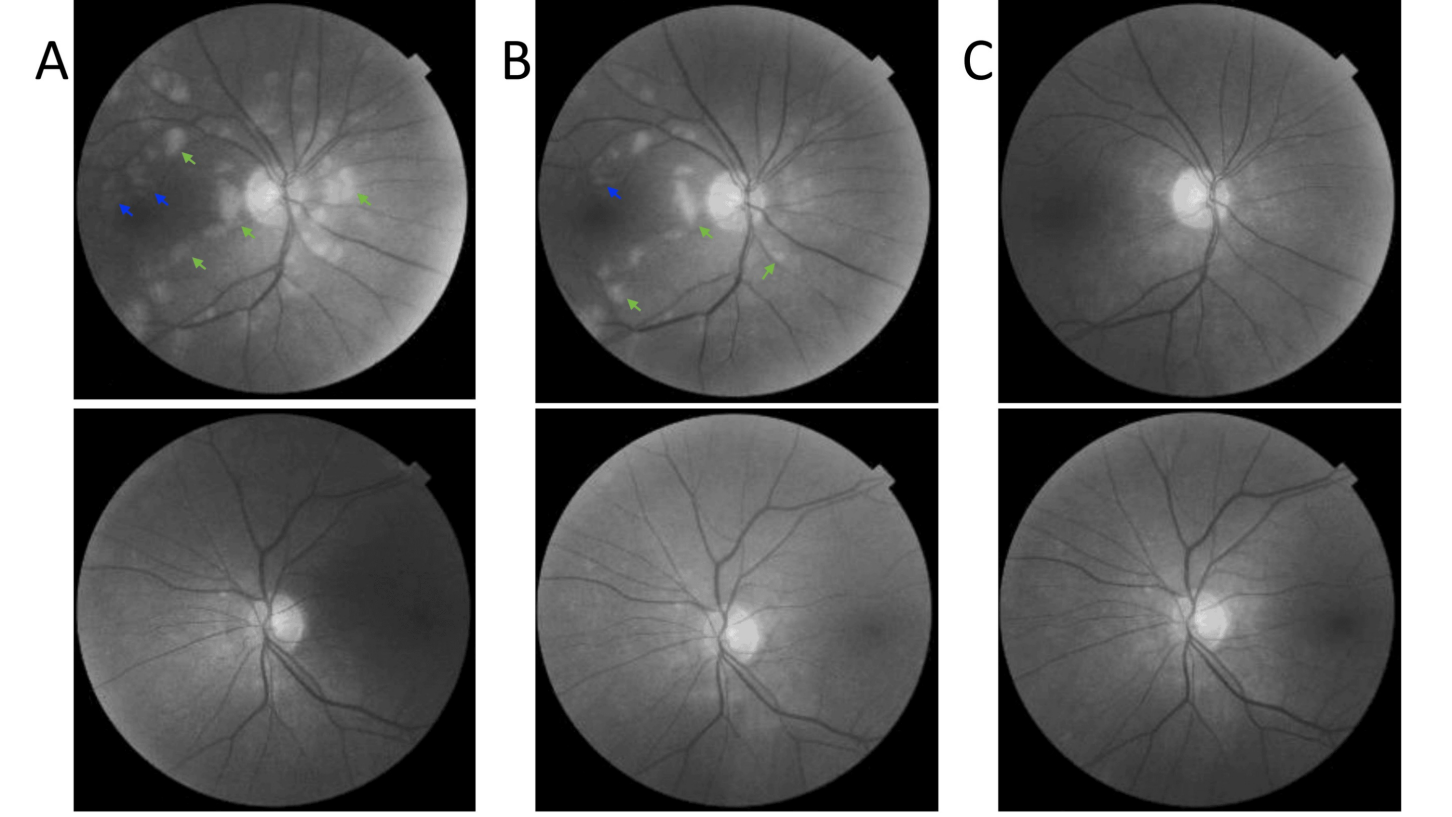
Series of red-free fundus images illustrating the evolution of retinal changes in the patient's eyes. Top row: A) Initial presentation – visible CWS (green arrows) and subtler parafoveal PF (blue arrows) changes around the macula. B) After two weeks – clinical improvement with a reduction in the number of CWS (green arrows) and gradual resolution of PF (blue arrows) changes. C) Postoperatively – complete resolution of retinal abnormalities. Bottom row: corresponding images of the left eye, showing no pathological changes on fundus examination.

**Figure 12:**
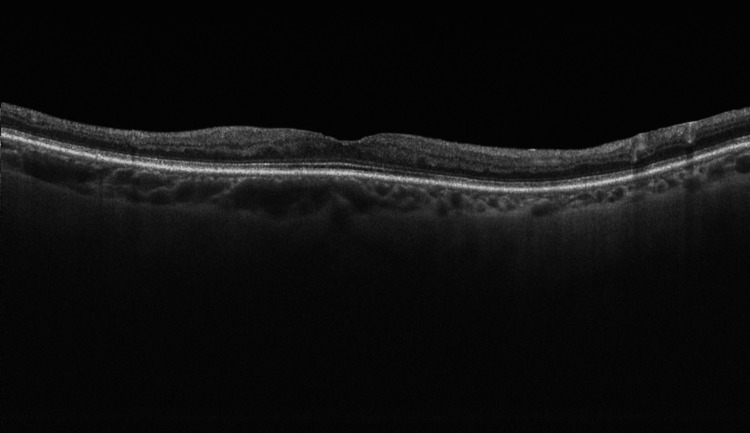
Optical coherence tomography scan of the macula of the right eye, obtained four weeks postoperatively and four months after the initial presentation. The scan shows visible thinning of the inner retinal layers and resolution of the swelling of the retinal nerve fibre layer (RNFL).

**Figure 13:**
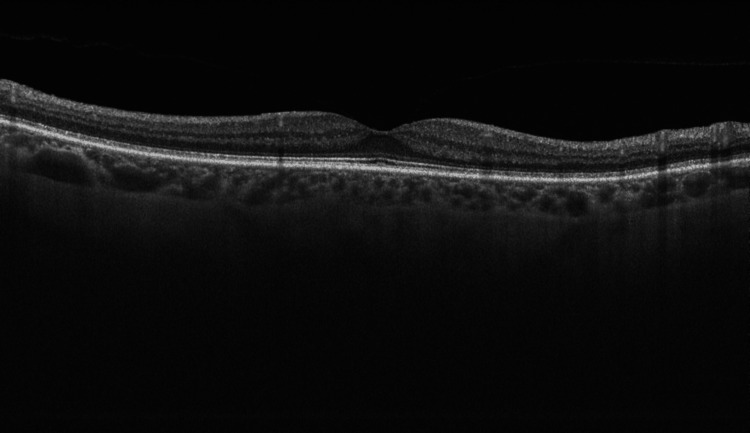
Optical coherence tomography scan of the macula of the left eye, obtained four weeks postoperatively and four months after the initial presentation. The OCT scan of the left eye shows normal macular architecture. The foveal contour is preserved, with normal layering of the retinal structures.

Four weeks postoperatively, the patient returned to the Ophthalmology Outpatient Clinic for a scheduled follow-up visit. She reported persistent semilunar-shaped dark spots in the visual field of the OD but denied any new visual disturbances since surgery. Due to reported visual field defects, Humphrey visual field testing was ordered. The examination revealed a scotoma involving the nasal visual field of the OD and disseminated sensitivity reduction in the same eye (Figure 10). Additionally, mild sensitivity reduction was observed in the visual field of OS. The findings in the OD visual field are consistent with the patient’s description. Despite the mild sensitivity reduction in OS, there are no retinal or optic nerve abnormalities in that eye that would account for these results, nor has the patient reported any subjective visual deterioration in OS. We believe that the visual field outcome for OS may be due to the fact that the patient underwent visual field testing for the first time in her life; however, these potential changes will be monitored during the subsequent scheduled visual field examinations. The BCVA in the OD improved to 0.5 logMAR, while, in the OS, it remained at 0.0 logMAR. In the Snellen near vision test performed with a +3.00 DS near addition, the visual acuity in the OD improved to 0.3 logMAR at 40 cm. Near vision in the OS was normal, corresponding to 0.00 logMAR at 40 cm. IOP in both eyes was normal. Fundus examination of the OD revealed the disappearance of Purtscher flecken (Figure 11), and OCT of the OD macula showed areas of inner retinal layer atrophy corresponding to their previous location (Figure 12). The examination of the posterior segment of the OS revealed no abnormalities (Figure 11), and OCT of the OS macula showed no pathology (Figure 13).

She remains under ophthalmologic and oncologic follow-up and awaits further oncological and endocrinological diagnostics due to a detected appendiceal neoplasm.

## Discussion

PLR is a rare diagnostic entity that may have been under-recognized for many years. Reviews on this topic emphasize that PF, considered pathognomonic for both PLR and PR, have been correctly recognized in only 25% of reported cases [3]. However, in recent years, the number of documented PLR cases has increased.

During a literature search, we found that about 100 PLR reports were published in PubMED between January 1, 2017, and April 3, 2025, compared to 146 cases indexed overall. This increase may be attributed, at least in part, to improved clinical awareness and broader access to diagnostic tools, particularly imaging techniques such as OCT. As associations between PLR and systemic conditions become more widely recognized, clinicians are increasingly likely to consider PLR in their differential diagnoses.

Our patient presented the CWS, the most prevalent manifestation of PLR. Although CWS are present in the vast majority of PLR cases, they are nonspecific and can be seen in many other retinopathies, such as diabetic and hypertensive retinopathies, but also in less common entities such as HIV retinopathy, cat scratch disease, SLE retinopathy, and others [7].

PF was present in the macular region of the affected eye of our patient. These changes are less frequent but considered pathognomonic. PF can be appreciated during the fundoscopy as areas of retinal whitening, with sharply demarcated borders, typically located in the peripapillary region. Unlike CWS, which are superficial and have ill-defined margins, PF shows a clear zone of approximately 50 µm on either side of retinal arterioles and venules, giving them a unique clinical appearance [3]. On OCT, PF can be visualized as structural alterations in the inner retinal layers. These include focal areas of increased hyperreflectivity predominantly involving the IPL, INL, OPL, and ONL [8]. These hyperreflective changes correspond anatomically to zones of retinal whitening seen clinically and represent areas of capillary nonperfusion and edema.

Our patient did not develop IRH - a finding similarly absent in approximately 35% of previously reported PLR cases [3]. PLR typically presents with bilateral retinal changes, yet in our case, the abnormalities were unilateral. The reason for this variation remains unclear, though PLR is generally associated with systemic conditions that could theoretically affect all small vessels.

Other, less common posterior segment findings in PLR include pseudo-cherry-red spot, macular oedema, and optic disc swelling [3]. Some patients may develop retinal neovascularisation due to retinal ischemia [9]. None of these features were observed in our patient. A systematic review by Serhan et al. indicated that CWS, IRH, PF, and optic disc swelling were associated with a good visual outcome. Prognostic factors associated with unfavourable outcomes remain unknown [2].

There is no consensus regarding the aetiology of PR and PLR. Available explanations are based on the formation of microemboli that occlude precapillary retinal arterioles [8]. These emboli may result from hyperviscosity due to elevated concentrations of acute-phase proteins, complement system activation, and increased leukocyte count during acute inflammation. While the initiating factor remains uncertain, the pathomechanism of PF and CWS is relatively well understood. Flow disruption in the small vessels of the retina leads to ischemia and consequent retinal oedema. As a result, swollen RNFL appears during fundoscopic examination as white CWS that obscure the retinal vessels. On OCT, these appear as thickening of the RNFL and ganglion cell layer (GCL). PF is thought to result from analogous changes in retinal layers located deeper than the RNFL and is seen on OCT as hyperreflectivity of the inner retinal layers. Since PF lies deeper, it does not obscure retinal vessels, which can be appreciated during the fundoscopic examination. These changes are generally transient; thickening and hyperreflectivity of the inner retinal layers typically diminish over several weeks or months [8].

Another clinical manifestation of PLR is paracentral acute middle maculopathy (PAMM). It is characterized by decreased visual acuity and visual field disturbances, such as central scotomas and grey spots, that reflect macular ischemia. Fundoscopic examination may be unremarkable or reveal white lesions in the macula. Two types of PAMM are recognized: type 1, involving the INL/OPL junction, and type 2, characterized by the OPL/ONL junction [10]. Macular changes in our patient were manifested as hyperreflectivity of inner nuclear layers and both type 1 and type 2 junctions. OCT findings in our patient are consistent with PAMM and PLR. Involvement of the INL/OPL junction suggests outer retinal ischemia at the level of the deep capillary plexus.

In a systematic review summarizing PR and PLR reports, Serhan et al. listed 68 PLR case reports. Seven of them were connected with malignancies: pancreatic and lung adenocarcinomas, cervical cancer, leukaemia, and multiple myeloma. These neoplasms are well-acknowledged risk factors for thromboembolic complications [11]. Additionally, a recent case by Teru et al. described PLR in a patient with acute ischemic colitis, highlighting that inflammatory abdominal conditions of non-neoplastic origin can also be associated with PLR [6]. While PLR secondary to inflammatory bowel disease, such as ischemic colitis, has been reported, to our knowledge, this is the first reported case linking PLR to ANEN or appendiceal inflammation.

ANENs are uncommon tumors and are found in approximately 0.2% of appendectomy specimens [12]. They are part of a broader group of neuroendocrine neoplasms (NENs) and include both indolent, highly differentiated neuroendocrine tumours (NETs) and aggressive, poorly differentiated neuroendocrine carcinomas (NECs). Their first manifestation may be acute appendicitis. In our case, histopathological examination following appendectomy for an appendiceal plastron revealed a well-differentiated grade 3 ANEN. Early-stage ANENs rarely cause systemic thromboembolic events, but may trigger regional complications such as mesenteric or splenic vein thrombosis. However, tumour-related metabolic activity can promote platelet aggregation and clot formation, contributing to a hypercoagulable state [13].

In our patient, elevated D-dimers were suggestive of such a prothrombotic state. This condition may have been further exacerbated by chronic inflammation due to the appendiceal plastron and long-standing smoking.

PLR is recognized as a self-limiting condition, and in many reported cases, spontaneous improvement of visual acuity and retinal findings occurs within weeks to months, even without systemic intervention [2]. In our view, the onset of PLR in this patient was temporally and causally linked to the evolving inflammatory process of the appendix, which was obstructed by a slowly developing ANEN. This process, we believe, initiated complement activation and leukoembolization, eventually leading to retinal microangiopathy typical for PLR. By the time of surgical intervention, the disease process had already reached its organizing stage, as evidenced by the CT scan, which revealed a perforated appendix forming an abscess and periappendiceal inflammatory complex (plastron).

Effective management of PLR depends on eliminating the underlying systemic cause. The most frequently administered therapy was systemic corticosteroids [2]. Although final BCVA appears comparable between patients receiving corticosteroids and those under observation alone, in cases involving optic nerve swelling, corticosteroids may offer some benefit [3]. If retinal neovascularisation is present, it must be treated to prevent further visual deterioration and retinal detachment. Available treatment methods include pan-retinal photocoagulation (PRP) and intravitreal anti-vascular endothelial growth factor (anti-VEGF) therapy.

## Conclusions

Although rare, PLR should be included in the differential diagnosis of retinal vascular disorders, particularly in patients presenting with systemic symptoms. This case underscores the importance of interdisciplinary collaboration in the timely identification of potentially life-threatening systemic conditions. To our knowledge, this is the first documented instance linking PLR with ANEN, thereby expanding the spectrum of systemic conditions associated with this retinal microvasculopathy. Further investigation is warranted to determine whether ANENs may contribute to microvascular injury through mechanisms such as tumor-related coagulopathy, neuroendocrine-mediated vasospasm, or immune complex deposition and to clarify the pathophysiological mechanisms of PLR to improve diagnostic and therapeutic strategies.

## References

[1] Tabandeh H, Rosenfeld PJ, Alexandrakis G, Kronish JP, Chaudhry NA (1999). Purtscher-like retinopathy associated with pancreatic adenocarcinoma. Am J Ophthalmol.

[2] Serhan HA, Abuawwad MT, Taha MJ (2024). Purtscher’s and Purtscher-like retinopathy etiology, features, management, and outcomes: a summative systematic review of 168 cases. PLoS One.

[3] Miguel AI, Henriques F, Azevedo LF, Loureiro AJ, Maberley DA (2013). Systematic review of Purtscher's and Purtscher-like retinopathies. Eye (Lond).

[4] Dyrda A, Matheu Fabra A, Aronés Santivañez JR, Blanch Rubio J, Alarcón Valero I (2015). Purtscher-like retinopathy as an initial presentation of iron-deficiency anaemia. Can J Ophthalmol.

[5] Lujan BJ, Coady PA, McDonald HR (2014). Spectral domain optical coherence tomography imaging of Purtscher-like retinopathy. Retin Cases Brief Rep.

[6] Teru S, Christensen CA, Brown J (2025). Purtscher-like retinopathy after acute ischemic colitis. J Vitreoretin Dis.

[7] Ioannides A, Georgakarakos ND, Elaroud I, Andreou P (2011). Isolated cotton-wool spots of unknown etiology: management and sequential spectral domain optical coherence tomography documentation. Clin Ophthalmol.

[8] Yaylali SA, Bromand N, Kilic G (2018). A new OCT finding in Purtscher-like retinopathy. Ophthalmic Surg Lasers Imaging Retina.

[9] Ramirez Marquez E, Mendez Bermudez IJ, Garcia N, Oliver AL (2023). Consequences of Purtscher-like retinopathy in a patient with systemic lupus erythematosus: a case report. Cureus.

[10] Sarraf D, Rahimy E, Fawzi AA (2013). Paracentral acute middle maculopathy: a new variant of acute macular neuroretinopathy associated with retinal capillary ischemia. JAMA Ophthalmol.

[11] Rubio-Jurado B, Sosa-Quintero LS, Guzmán-Silahua S, García-Luna E, Riebeling-Navarro C, Nava-Zavala AH (2021). The prothrombotic state in cancer. Adv Clin Chem.

[12] Gobishangar S, Gobinath S, Thevamirtha C, Sarmila S, Kasthuri S, Paramanathan S (2023). Prevalence of neuroendocrine tumours (NET) in patients undergoing appendicectomy for acute appendicitis: a tertiary care study. Cureus.

[13] Bachelani AM (2022). Mesenteric venous thrombosis: a rare complication of small bowel neuroendocrine tumor presenting with gangrenous appendicitis. J Surg Case Rep.

